# Christmas Day 1914

**Published:** 1914-12-19

**Authors:** 


### The Hospital

The Workers' Newspaper of Administrative Medicine and Institutional Life, Administration, National Insurance and Health.

No. 1487, Vol. LVII. SATURDAY, DECEMBER 19, 1914. PRICE ONE PENNY.

### CHRISTMAS DAY 1914.

Christmas this year should live in the memory of all hospital workers throughout the Empire. Those workers, from the highest to the lowest, have had their labours materially added to as a direct consequence of this war. At the outset work in the principal hospitals throughout the United Kingdom was disturbed and interfered with by the drafts made upon the working staff, from which have been taken doctors, nurses, orderlies, officers, sailors, and soldiers, whose duties have necessitated their devoting the whole or a large portion of their time to matters incidental to the war. As the war progressed further stress was caused by the bringing into working order of the Territorial Hospital system throughout the country, and continuously there has been the drain due to the call for an increasing number of workers to make good vacancies and to meet the ever-growing need for trained workers at the Front. War-time has so proved a period of ever-increasing self-denial, growing strain, and never-ending burden for all hospital workers whose duty it has been to minister to the wounded and the sick. That is why this season and the months which have preceded it must prove full of glorious memories to all such workers throughout the Empire. For them Christmas this year will have a very real and special meaning, for they have spent themselves willingly and heartily in personal service, and have so proved worthy of the noble calling which they habitually follow.

All voluntary hospital workers during 1914 should have good cause to thank God and take courage. The organisation of the Territorial Hospital system, the preparation of additional wards, the meeting of special demands for money, and the anxieties and additional labour these have entailed upon chairmen of committees, treasurers, committeemen, hospital officers, and the members of the lay staff, have been great indeed. It is memorable and glorious that all this extra service has been rendered gladly with a patriotism and self-denial, combined with an expeditious efficiency, which could not have been surpassed. Britons pride themselves on their voluntary hospital system, and to that pride may be added reverence when the war is over, for the teaching and spirit of the voluntary hospital have made their weight felt for good, not only amongst the workers, but amongst the patients too. All classes have been fired by the spirit of the hospitals through the war, and the women, and for that matter the men also, who may join the Voluntary Hospitals Brigade, for which upwards of 100 persons have already sent us offers of service, will so be enabled to take their individual share of the work of ministering to the wounded. Who can start the year better than by sending his or her name to the Editor as a member of this Brigade? We offer to all our readers, and especially to all workers among the sick, hearty Christmas greetings. May they each have a few moments to realise the work they have been privileged to do for the sick and wounded! There is nothing that enables a man or woman to learn how to live like personal service in one of the great causes which The Hospital has the privilege to represent.

